# Postural change in volunteers: sympathetic tone determines microvascular response to cardiac preload and output increases

**DOI:** 10.1007/s10286-015-0286-x

**Published:** 2015-08-18

**Authors:** Eva Klijn, Sjoerd Niehof, A. B. Johan Groeneveld, Alexandre Pinto Lima, Jan Bakker, Jasper van Bommel

**Affiliations:** Department of Intensive Care, Erasmus MC University Medical Center, PO box 2040, Room H 621, 3000 CA Rotterdam, The Netherlands; Department of Anesthesiology, Erasmus MC University Medical Center, Rotterdam, The Netherlands

**Keywords:** Microcirculation, Head up tilt, Passive leg raising, Cardiac output, Sympathetic tone

## Abstract

**Purpose:**

Microvascular perfusion may be a non-invasive indicator of fluid responsiveness. We aimed to investigate which of the microvascular perfusion parameters truly reflects fluid responsiveness independent of sympathetic reflexes.

**Methods:**

Fifteen healthy volunteers underwent a postural change from head up tilt (HUT) to the supine position, diminishing sympathetic tone, followed by a 30° passive leg raising (PLR) with unaltered tone. Prior to and after the postural changes, stroke volume (SV) and cardiac output (CO) were measured, as well as sublingual microcirculatory perfusion (sidestream dark field imaging), skin perfusion, and oxygenation (laser Doppler flowmetry and reflectance spectroscopy).

**Results:**

In responders (subjects with >10 % increase in CO), the HUT to supine change increased CO, SV, and pulse pressure, while heart rate, systemic vascular resistance, and mean arterial pressure decreased. Additionally, microvascular flow index, laser Doppler flow, and microvascular hemoglobin oxygen saturation and concentration also increased.

**Conclusion:**

When preload and forward flow increase in association with a decrease in sympathetic activity, microvascular blood flow increases in the skin and in the sublingual area. When preload and forward flow increase with little to no change in sympathetic activity, only sublingual functional capillary density increases. Therefore, our results indicate that sublingual functional capillary density is the best parameter to use when evaluating fluid responsiveness independent of changes in sympathetic tone.

## Introduction

In the critically ill, non-invasive microcirculatory perfusion abnormalities measured sublingually or cutaneously are associated with a poor outcome, relatively independent of global hemodynamics [1]. They can be ameliorated by fluid infusion and, in fact, perfusion abnormalities may be surrogate indicators of fluid responsiveness, i.e., an increase in cardiac output (CO)/stroke volume (SV) with cardiac preloading, suggesting that systemic blood flow still feeds the microcirculation in the critically ill [2, 3]. Sympathetic activation resulting in peripheral vasoconstriction may partly explain the sometimes observed discrepancy between global and microvascular hemodynamics in the critically ill [4, 5]. Changes in sympathetic tone concomitant with changes in volemic status (e.g., decreasing sympathetic tone by fluid infusion) may thus affect how microvascular perfusion reflects global hemodynamics.

Because of the potential detrimental effects of fluids in critically ill patients, the concept of fluid responsiveness is an important guide for fluid administration. Fluid does not necessarily increase SV and CO, as explained by the curve-linearity of the Frank–Starling curve. When the heart is operating on the steep part of the curve, an increase in preload results in an increase in stroke volume (i.e., responders). If the heart is operating on the distal part of the curve, an increase in preload will not result in an increase in SV (i.e., non-responders).

Head up tilting (HUT) is used in healthy volunteers and patients with vagal syncope to diminish preload and activate the sympathetic nervous system, so that reverse positioning is expected to lower sympathetic activity [6]. The head up tilt-to-supine position induces a large preload challenge as during the postural change approximately 500 ml of pooled blood redistributes to the central circulation [7, 8]. The passive leg raising (PLR) test is a frequently used test in clinical practice, which also induces a preload challenge due to redistribution of blood; however, in a smaller amount (150–300 ml) [9, 10]. The PLR test may not alter sympathetic activity in sedated patients; or it may do so, but to a much lesser degree than HUT. The postural change may result in small alterations of carotid and baroreceptor positioning and is more likely to increase sympathetic activity. Comparing the two maneuvers in microvascular perfusion parameters may thus give insight into the true parameters associated with fluid responsiveness independent of alterations in sympathetic tone.

We hypothesized that the site and type of microvascular perfusion response to a cardiac preload challenge depends on sympathetic tone alterations, e.g., peripheral vasodilation with diminished activity. Therefore, in the current study, we focused on the microvascular effects of increasing preload with and without altering sympathetic activity in healthy volunteers. We aimed to investigate which of the microvascular parameters can be used to truly reflect a preload challenge and resultant increase in forward flow (so-called fluid responsiveness) by studying the effects of two postural changes known to establish a preload challenge, but with different effects on the autonomic nervous system. We used the sublingual and cutaneous microvascular beds because these are the most easily accessible and most studied microvascular beds in critically ill patients.

## Patients and methods

### Subject population

This study was performed in the Erasmus MC University Medical Center (Rotterdam, The Netherlands) and was approved by the local ethics committee. Written informed consent was obtained in each case. We studied 15 healthy volunteers [nine men, six women; median age 27 years (23–28.5)] with no history of cardiovascular disease or the use of any vaso-active medication. The phase of the menstrual cycle of the female subjects at the time of the study was not recorded. Subjects were recruited from the Erasmus University of Rotterdam.

### Protocol

**Fig. 1:**
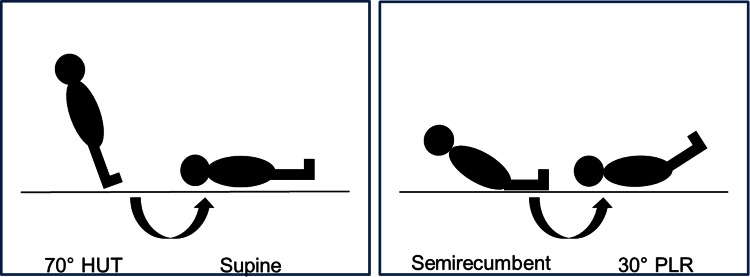
Postural changes. The 70° head up tilt (HUT)-to-supine posture change induces a large preload challenge, which redistributes approximately 500 mL of pooled blood to the central circulation. The semi-recumbent position to 30° passive leg raising (PLR) induces a smaller preload challenge, which redistributes approximately 150–300 mL of pooled blood to the central circulation

Study measurements took place in a quiet, temperature-controlled room and were performed in four stages. Subjects were comfortably restrained on an electric tilt table with footplate support. Following the positioning of the non-invasive recorders, subjects underwent a passive head up tilt to an angle of 70° and remained tilted for 5 min, after which baseline measurements were made. Subjects were returned to the supine position (0°) and measurements were repeated after 5 min. Subjects were placed in a regular hospital bed, and were positioned in a semi-recumbent position of 30°, all measurement equipment was recalibrated, and measurements were made. Secondly, a PLR test was performed, resulting in a 0° supine position of the thorax with the legs elevated 30° for 5 min, as described previously, and measurements were repeated [11]. The different positions are demonstrated in Fig. 1. During these postural changes, both hands (one used to measure peripheral perfusion and the other used to measure global hemodynamics) were passively immobilized at heart level using a sling.

### Measurements of systemic hemodynamics

Global hemodynamic parameters included heart rate (HR), CO, SV, mean arterial pressure (MAP), and pulse pressure (PP), which were continuously measured non-invasively with a Finometer^®^ (Finapres Medical System, Amsterdam, The Netherlands). The Finometer gives waveform measurements similar to intra-arterial recordings and computes beat-to-beat hemodynamic parameters, including CO and SV. The Finometer measures brachial pressure and corrects for finger pressure accordingly. Finger arterial pressure was measured using a finger cuff (on the left hand index finger) in combination with an infrared plethysmograph consisting of a light source (infrared light-emitting diode) and a light detector (infrared photodiode) [12]. Systemic vascular resistance (SVR) was calculated using the following formula: SVR = MAP/CO × 80 (dyn s cm^−5^).

### Sidestream dark field imaging (SDF)

Sublingual microvascular blood flow was evaluated using SDF (MicroVision Medical, Amsterdam, The Netherlands). Image acquisition and subsequent analyses were performed according to published consensus criteria [13]. In brief, after the removal of saliva with gauze, the device was gently applied to the sublingual area by investigators well trained in SDF imaging. For each stage, five 20 s sequences were recorded, each from different adjacent areas. The sequences were stored under a random number and later analyzed according to the recent consensus with dedicated software (Microcirculatory Analysis Software (MAS 3.0) Academic Medical Center, Amsterdam). Microvascular flow index (MFI) was calculated after dividing each image into four equal quadrants. Quantification of flow was determined using an ordinal scale (0, no flow; 1, intermittent flow; 2, sluggish flow; 3, normal flow; and 4, hyperdynamic flow) [14]. MFI is the average score of all quadrants for a given time point. Vessel density was calculated, according to the consensus, in two manners. First, functional capillary density (FCD) was calculated by measuring the total length of perfused capillaries divided by the image area. Second, vessel density (VD) was calculated by inserting a grid of three equidistant horizontal and three equidistant vertical lines over the image. VD is equal to the number of vessels crossing these lines divided by their total length. Flow was then categorized as present, intermittent, or absent, allowing calculation of the proportion of perfused vessels (PPV). To determine the intrarater variability of the sublingual microvascular parameters, the complete image analysis on 90 SDF sequences was repeated at a later time point. The intrarater variability of the SDF analysis was determined by calculating an intraclass correlation coefficient on consistency, considered good when ≥0.6. The intraclass correlation for MFI was 0.75, for FCD 0.72, for VD 0.73, and for PPV 0.93.

### Laser Doppler flowmetry (LDF) and reflection spectrophotometry (RS)

LDF and RS were performed using an O2C device (Oxygen to See, LEA Medizintechnik GmbH, Giessen, Germany). The tissue was illuminated with a pulsed 830-nm class 1 laser diode and the backscattered light was spectrally analyzed to assess the velocity-dependent frequency shifts caused by flowing red blood cells. The microvascular hemoglobin oxygen saturation (µHbSO_2_) and relative hemoglobin concentration (rHb) were measured by illuminating tissue with visible white light (500–630 nm), which is backscattered and changed in color according to its O_2_ saturation. The mean flow and µHbSO_2_ were recorded and averaged over a steady-state period of 1 min.

### Statistical analysis

Statistical analyses were performed in SPSS (version 19.0, SPSS, Chicago, IL, USA). Non-parametric tests were used because of the relatively small numbers, even though the variables were mostly normally distributed. Intragroup comparisons were done with the help of the Wilcoxon matched pairs test. Comparison between groups was done with a Mann–Whitney *U* test. To correct for the different magnitudes in the change of CO during the HUT to the supine position and PLR, the data were normalized for the change in CO. Therefore, the changes in the parameters of microvascular perfusion during the HUT to the supine position or PLR were divided by the changes in CO during the corresponding postural change. To test if the changes in the parameters of microvascular perfusion corrected for CO differed between the two postural changes, we compared the CO-normalized changes in parameters of microvascular perfusion during HUT to supine with those same changes during PLR using a Wilcoxon matched pairs test. We defined fluid responsiveness by an increase in CO ≥10 and 5 % for HUT to supine and PLR, respectively, because of their difference in preload augmentation. In critically ill patients, fluid responsiveness is defined as an increase in CO of >10 % following a fluid bolus of 500 mL. We therefore used this cut-off value for HUT to supine, as approximately 500 mL of blood is redistributed to the central compartment [15], whereas about 250 mL is recruited during PLR [9, 10]. Data are summarized by mean ± standard deviation. A *P* < 0.05 was considered statistically significant. Exact *P* values are given.

## Results

### Effects of HUT to the supine position

**Fig. 2:**
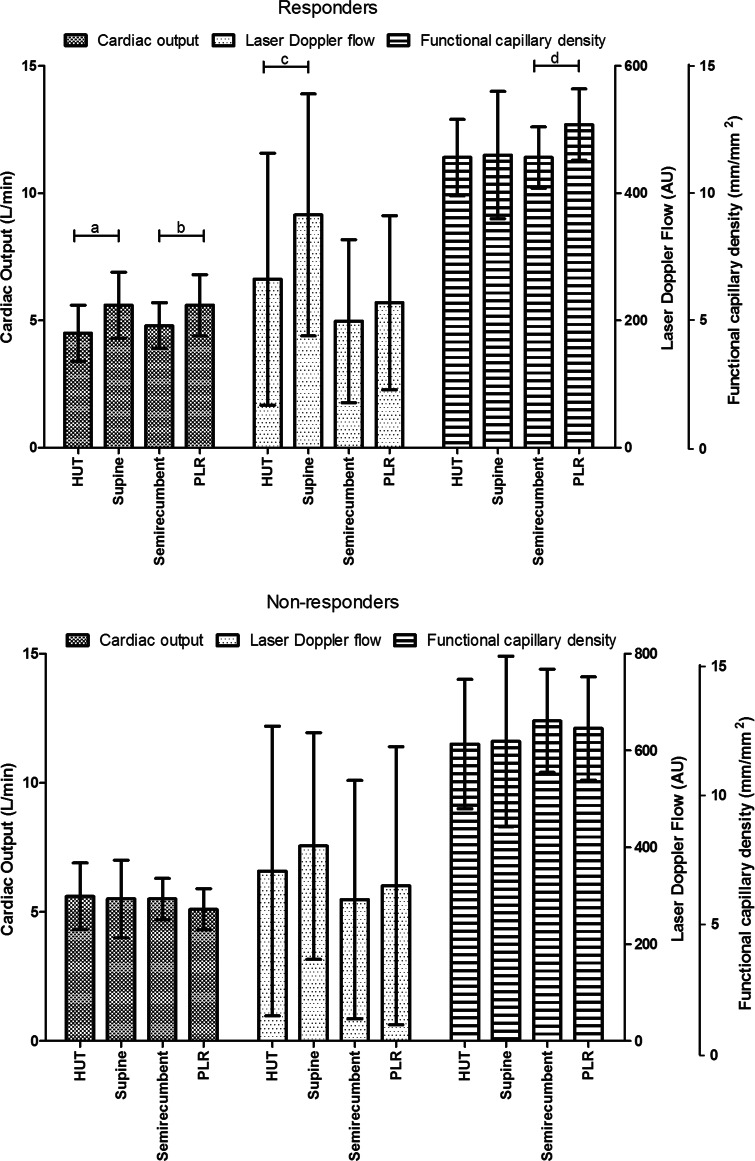
Changes in cardiac output (CO), laser Doppler flow (LDF), and functional capillary density (FCD) during HUT to the supine position and PLR posture change. The *upper panel* represents the results for the responders and the *lower panel* for the non-responders. (*a* *P* = 0.002, *b* *P* = 0.027, *c* *P* = 0.003, *d* *P* = 0.046). Mean ± standard deviation

Table 1 shows global hemodynamics and microvascular perfusion values during HUT to the supine position in responders (*n* = 12), defined by a ≥10 % increase in CO, and non-responders (*n* = 3). There were no differences in hemodynamic and microvascular perfusion parameters between responders and non-responders in the HUT position. The change from HUT to the supine position increased CO, SV, and PP, while HR, SVR, and MAP decreased in the responders. Additionally, MFI, LDF, µHbSO_2_, and rHb increased. In the non-responders, systemic hemodynamics, as well as regional tissue perfusion parameters, did not change. The change in SVR following HUT to the supine position differed between responders and non-responders (*P* = 0.021). Figure 2 shows the changes in CO, LDF, and FCD during the HUT to the supine position in responders and non-responders.

**Table 1:** Hemodynamics and microvascular perfusion values during HUT and supine position, in responders (R) and non-responders (NR) based on a ≥10 % increase in cardiac output to the posture change

	Responders (*n* = 12)			Non-responders (*n* = 3)		
	HUT	Supine	*P*_1_	HUT	Supine	*P*_2_
Heart rate (bpm)	78 ± 7	67 ± 8	0.002	77 ± 4	61 ± 3	0.11
Stroke volume (ml)	59 ± 16	85 ± 20	0.002	73 ± 14	91 ± 22	0.11
Mean arterial pressure (mmHg)	107 ± 15	96 ± 8	0.028	104 ± 15	103 ± 15	0.66
Pulse pressure (mmHg)	44 ± 4	48 ± 5	0.023	46 ± 8	50 ± 10	0.11
Systemic vascular resistance (dyn s cm^−5^)	2049 ± 772	1438 ± 384	0.002	1523 ± 268	1555 ± 348	1.00
Microvascular flow index (AU)	2.8 ± 0.4	3.0 ± 0	0.034	3.0 ± 0	3.0 ± 0	0.32
Vessel density (1/mm)	9.2 ± 1.1	9.9 ± 0.6	0.099	8.9 ± 2.1	9.7 ± 1.8	0.11
Proportion of perfused vessels (%)	95 ± 9	100 ± 0.5	0.063	100 ± 0	100 ± 0	1.0
µHbSO_2_ (%)	65 ± 10	70 ± 9	0.009	70 ± 16	77 ± 5	0.11
rHb (AU)	37 ± 8	46 ± 7	0.005	48 ± 11	57 ± 13	0.29

Mean ± standard deviation

*P*_*1*_ HUT vs supine position in R, *P*_*2*_ HUT vs supine position in NR, *µHbSO*_*2*_ microvascular hemoglobin oxygen saturation, *rHb* relative hemoglobin concentration, *AU* arbitrary units

### Effects of PLR

Table 2 shows the global hemodynamic and microvascular perfusion values during the PLR in responders (*n* = 6), defined by a ≥5 % increase in CO, and non-responders (*n* = 9). There were no differences in hemodynamic and microvascular perfusion parameters between responders and non-responders in the semi-recumbent position. During the PLR, CO, SV, and FCD increased in responders, while SVR decreased. In the non-responders, systemic hemodynamic and microvascular perfusion parameters remained unaltered. The change in SV (*P* = 0.001), SVR (*P* = 0.001), and FCD (*P* = 0.018) during PLR differed between responders and non-responders. Figure 2 shows the changes in CO, LDF, and FCD during the PLR in responders and non-responders. An increase in CO of ≥5 % to a PLR did not predict responsiveness during HUT to the supine position postural change (sensitivity 42 %, specificity 66 %), suggesting that the preload effect of the two maneuvers on CO was modulated by different effects on afterload and/or contractility.

**Table 2:** Hemodynamics and microvascular perfusion values during passive leg raising (PLR) in responders (R) and non-responders (NR) based on a ≥5 % increase in cardiac output in response to PLR

	Responders (*n* = 6)			Non-responders (*n* = 9)		
	Baseline	PLR	*P*_1_	Baseline	PLR	*P*_2_
Heart rate (bpm)	64 ± 6	66 ± 10	0.75	65 ± 9	65 ± 11	0.17
Stroke volume (ml)	76 ± 15	85 ± 15	0.028	85 ± 13	81 ± 17	0.21
Mean arterial pressure (mmHg)	99 ± 11	96 ± 9	0.25	96 ± 8	91 ± 9	0.051
Pulse pressure (mmHg)	45 ± 6	45 ± 6	0.35	45 ± 8	42 ± 8	0.09
Systemic vascular resistance (dyn s cm^−5^)	1716 ± 494	1430 ± 348	0.030	1429 ± 170	1443 ± 189	0.37
Microvascular flow index (AU)	3.0 ± 0.1	3.0 ± 0.1	1.0	3.0 ± 0	3.1 ± 0.1	0.08
Vessel density (1/mm)	9.5 ± 1.0	10.1 ± 1.4	0.12	10.0 ± 1.3	9.9 ± 1.2	0.68
Proportion of perfused vessels (%)	100 ± 0	100 ± 0	1.0	99.7 ± 0.9	99.6 ± 1.3	0.66
µHbSO_2_ (%)	65 ± 9	68 ± 11	0.46	68 ± 12	67 ± 14	0.52
rHb (AU)	39 ± 8	44 ± 5	0.12	40 ± 7	39 ± 9	0.77

Mean ± standard deviation

*P*_*1*_ baseline vs PLR in R, *P*_*2*_ baseline vs PLR in NR, *µHbSO*_*2*_ microvascular hemoglobin oxygen saturation, *rHb* relative hemoglobin concentration, *AU* arbitrary units

### Global hemodynamics with HUT to the supine position vs PLR

The increase in CO and SV were greater with HUT to the supine position than with PLR (*P* = 0.008); the decrease in HR (*P* = 0.001) and SVR (*P* = 0.006) were greater, too. In responders to PLR, the decrease in HR was greater with HUT to the supine position than with PLR (*P* = 0.028), with a similar increase in CO.

### Microvascular effects normalized for CO

Table 3 shows the changes in microvascular perfusion values adjusted for the change in CO during HUT to the supine position and PLR in responders (*n* = 12) and non-responders (*n* = 3) based on a ≥10 % increase in CO to the postural change from HUT to the supine position. In responders, the change (adjusted for CO) in the microvascular flow index increased during HUT to the supine position as compared to PLR. In responders, there is a trend to an increased functional capillary density during PLR as compared to the HUT to the supine position maneuver.

**Table 3:** Changes (Δ) in microvascular perfusion values adjusted for change in cardiac output (CO) during head up tilt to supine (HUT) and passive leg raising (PLR) (e.g., change in microvascular flow index during HUT divided by change in CO during HUT) in responders (R) and non-responders (NR) based on a ≥10 % increase in CO in response to the posture change from HUT to supine

	Responders HUT (*n* = 12)			Non-responders HUT (*n* = 3)		
	HUT	PLR	*P*_1_	HUT	PLR	*P*_2_
ΔMicrovascular flow index (AU)	0.24 ± 0.42	−0.08 ± 0.24	0.037	−0.1 ± 0.23	0.9 ± 1.6	0.18
ΔFunctional capillary density (mm/mm^2^)	−0.09 ± 3.0	6.4 ± 22	0.060	−7.5 ± 13.5	3.0 ± 3.3	0.29
ΔVessel density (1/mm)	0.61 ± 1.4	1.2 ± 3.1	0.43	2.3 ± 5.1	−7.5 ± 5	0.11
ΔProportion of perfused vessels (%)	5.7 ± 11.1	−1.1 ± 5.6	0.18	0 ± 0	0 ± 0	1.0
ΔLaser Doppler flow (AU)	96 ± 94	241 ± 616	0.75	341 ± 323	−196 ± 277	0.11
ΔµHbSO_2_ (%)	6 ± 8	36 ± 115	0.81	36 ± 55	−4 ± 20	0.11
ΔrHb (AU)	10 ± 8	3 ± 40	0.75	109 ± 96	−7 ± 29	0.11

Mean ± standard deviation

*P*_*1*_ changes HUT vs changes PLR in R, *P*_*2*_ changes HUT vs changes PLR in NR, *µHbSO*_*2*_ microvascular hemoglobin oxygen saturation, *rHb* relative hemoglobin concentration, *AU* arbitrary units

## Discussion

Our proof-of-principle study suggests that, in healthy subjects, the two postural maneuvers (HUT to the supine position and PLR) are not comparable in their effects on systemic and microvascular perfusion variables. During postural change from HUT to the supine position, cardiac preload and forward flow increase along with decreased sympathetic activity. This is evidenced by a greater decrease in heart rate and vascular resistance than that evoked by PLR. Microvascular perfusion increases (i.e., blood flow to the skin), as well as blood flow to the sublingual area. During PLR, cardiac preload and forward flow increase without (or with only mildly altered) sympathetic activity. This is evidenced by an unchanged heart rate and a small decrease in systemic vascular resistance; only sublingual functional capillary density increases. Therefore, our results indicate that sublingual functional capillary density is the best parameter to use when evaluating fluid responsiveness independent of alterations in sympathetic tone.

The HUT maneuver has been extensively used to study orthostatic hypotension and autonomic failure [6, 16]. There is only scarce data on microcirculatory perfusion during HUT, however. A recent study in children using near infrared spectroscopy demonstrated a decrease in regional tissue oxygenation in the splanchnic region when the subject was tilted [17]. A study measuring femoral, as well as brachial, blood flow using ultrasound Doppler demonstrated a decrease in blood flow in the upper and lower limbs during a 60° HUT [8].

The differences in systemic hemodynamic and microvascular perfusion responses following the HUT to supine postural change and PLR test could be explained by the fact that, initially, HUT leads to a decreased blood volume in the central circulation, and baroreflex-mediated increases in peripheral vascular resistance help to maintain arterial blood pressure. These changes are then reversed during subsequent repositioning [8, 18, 19]. The difference in HR and SVR response between the HUT to supine posture change and the PLR underlines the fact that sympathetic activity decreases following the HUT to supine posture change and exhibits little change during the PLR. This differential effect may also explain the imperfect overlap in cardiac output responses to HUT to supine and PLR maneuvers.

The HUT serves as a model for central hypovolemia, while in a semi-recumbent position the subject is supposed to be normovolemic. The preload challenge from HUT to supine was of larger magnitude than the PLR test, explaining the differences in effect on hemodynamic as well as regional tissue perfusion parameters. We therefore studied microvascular responses for equal changes in CO responses. These data show that when the microvascular perfusion parameters are normalized for the changes in CO, there is still a trend in increase in sublingual functional capillary density during the PLR compared with the HUT to supine.

Although this was a proof-of-principle study, several limitations need to be addressed. First, we used a low number of study subjects, making it difficult to study potential gender differences and subgroup comparisons, especially in the HUT to the supine position postural change where the number of non-responders was low. Additionally, our study population consisted of young subjects; thus, it is not clear if our results can be extrapolated to an older population. Third, we have not accounted for the possibility of relative hypovolemia: we assumed our study population was normovolemic due to normal fluid intake by every subject prior to the start of the study.

## Conclusion

When preload and forward flow increase in association with decreased sympathetic activity, microvascular blood flow increases in the skin and in the sublingual area. When preload and forward flow increase with little to no change in sympathetic activity, as seen during the PLR test, sublingual functional capillary density is increased. Therefore, our results indicate that sublingual functional capillary density is the best parameter to use when evaluating fluid responsiveness independent of the reflex activity of the autonomic nervous system.
